# Salivary film thickness and MUC5B levels at various intra-oral surfaces

**DOI:** 10.1007/s00784-022-04626-3

**Published:** 2022-08-09

**Authors:** Z. Assy, D. H. J. Jager, H. S. Brand, F. J. Bikker

**Affiliations:** 1 Department of Oral Biochemistry, Academic Centre for Dentistry Amsterdam, University of Amsterdam, VU University Amsterdam, Amsterdam, The Netherlands; 2 Department of Oral and Maxillofacial Surgery/Oral Pathology, Amsterdam UMC and Academic Center for Dentistry (ACTA), Vrije Universiteit Amsterdam, Amsterdam Institute for Infection and Immunity, Amsterdam, The Netherlands

**Keywords:** Salivary film thickness, Salivary secretions, MUC5B level, Palatal surface area, Sialopapers

## Abstract

**Objectives:**

In this study, we investigated the salivary film thickness and the MUC5B levels at various intra-oral locations in healthy volunteers, with a focus on the palate. Besides, measurements of the palatal surface area were included to explore the possible relationships between the palatal surface area and the palatal salivary film and MUC5B levels.

**Materials and methods:**

The salivary film thickness was determined using filter strips, which were pressed to the mucosal surfaces of five different intra-oral locations; conductance was then analysed using a Periotron. After elution of the strips, the MUC5B levels at various intra-oral locations were determined using ELISA. The palatal surface area was measured using an intra-oral scanner. The surface area was subsequently calculated using the software.

**Results:**

The anterior tongue had the thickest salivary film and also the highest levels of MUC5B, while the anterior palate had the thinnest salivary film and lowest MUC5B levels. There was no association between the palatal surface area and the salivary film thickness of the palate.

**Conclusion:**

The salivary film and MUC5B levels are unequally distributed over the intra-oral regions of the soft tissues. The lack of association between the palatal surface area and the salivary film thickness indicates that a larger surface area is not associated with a relative thinner palatal salivary film.

**Clinical relevance:**

The results of the current study increase our understanding of saliva distribution in the oral cavity and could be used as reference values for future studies.

## Introduction

The salivary glands produce saliva which contains a wide range of proteins and ions [1]. After secretion, and facilitated by swallowing, saliva is spread over the hard and soft tissues in the oral cavity as a thin salivary film [2, 3]. A major compound of this salivary film is MUC5B, a large glycoprotein with a wide variety of hydrophilic carbohydrate side chains [4]. MUC5B plays a crucial role in saliva’s water-retaining properties, such as moistening, visco-elasticity and lubrication [3, 5]. As a consequence, an impaired flow rate, *i.e.* hyposalivation, leads to lower availability of both water and salivary proteins and to the insufficient replenishing of the intra-oral salivary film [6]. Subsequently, this leads to impaired mucosal moistening and clinical problems, such as difficulties with speech and swallowing, pain and xerostomia [7, 8].

It was recently shown that the severity of xerostomia varied at different intra-oral locations [9, 10]. In particular, it was found that the perceived oral dryness was most profound for the (posterior) palate. Hypothetically, this could be related to an impaired salivary film and reduced MUC5B content, especially at the palate.

In the past, multiple studies have investigated the salivary film thickness including the total protein concentration at various mucosal surfaces [11–14]. These studies found that the total protein concentration displayed a wide variation depending on its location [12–14]. The protein concentration showed a negative correlation with the salivary film thickness, indicating that decreased salivary films were related to increased protein concentrations [12–14]. These findings reveal that the protein levels in the film are mainly influenced by the film volume, but did not provide detailed insights into the protein composition at various intra-oral locations. Determination of MUC5B levels in the salivary film could help to increase our understanding of salivary distribution in the oral cavity.

To understand the distribution of saliva over various surfaces, it is also important to measure the intra-oral surface areas. The dimensions of the intra-oral surface areas have previously been analysed in order to determine the distribution and average thickness of the salivary film covering the teeth and oral mucosa [15–17]. Especially the palate plays a major role in xerostomia because the salivary film thickness at the anterior part of the palate is relatively thin compared to other intra-oral surfaces [2, 11–14, 18–21]. Besides, the central part of the anterior palate is devoid of minor salivary glands [22]. Therefore, in order to increase our understanding of the distribution of the salivary film, measurements of the palatal surface area were included in the current study. It is envisaged that these measurements could serve as a reference for future studies, *e.g.* on salivary film integrity related to various oral diseases.

Therefore, the present study aims to determine the salivary film thickness and the MUC5B levels at various intra-oral locations in healthy volunteers. Furthermore, we included measurements of the palatal surface area to explore the possible relationships between the palatal surface area and the palatal salivary film thickness and MUC5B levels. We hypothesised that healthy individuals with comparable salivary flow rates, but differences in palatal surface area will have a different distribution of the salivary film and/or the MUC5B levels; individuals with a larger palatal surface area would have a thinner salivary film at the palate and also less availability of MUC5B.

## Materials and methods

### Participants

The study was approved by the Ethics Review Committee at the Academic Center for Dentistry Amsterdam (ACTA; 202065). Volunteers were recruited at ACTA through posters. Eligibility criteria required volunteers to be 18 years or older, preferably without having the tendency to gag. Informed written consent was obtained from all volunteers. No personal data of volunteers were recorded, with the exception of age and sex. Volunteers using polypharmacy (more than four medications) or specific xerogenic medications were excluded for saliva collection. Xerogenity of the medications was determined using the medication guides published by Sreebny and Schwartz (1986), Wolff et al. (2016) and the Dutch Pharmacotherapeutic Compass [23–25]. The reporting of this study conforms to the STrengthening the Reporting of OBservational studies in Epidemiology (STROBE) statement [26].

### Study variables

#### Subjective oral dryness assessment

The Xerostomia Inventory (XI) was used to measure the overall dry-mouth experience. The XI consists of 11 items on a 5-point Likert scale ranging from 1 = “never” to 5 = “very often.” The items are about oral dryness and mouth feel. Participants indicate on each item how often they suffer from problems with regard to mouth feel and oral dryness. The scores of the 11 items are summed, resulting in a total XI-score that ranges between 11 (no xerostomia) and 55 (extreme xerostomia) [27].

In addition, participants completed the Regional Oral Dryness Inventory (RODI) to measure the intra-oral perceived dryness [8, 9]. This questionnaire contains 9 schematic illustrations of different locations in the oral cavity. Four illustrations represent areas in the upper jaw: the upper lip, anterior part of the palate (including the rugae), inside part of the cheeks and posterior part of the palate (from the rugae up to the end of the soft palate). Four illustrations represent areas in the lower jaw: the lower lip, floor of the mouth, posterior part of the tongue (from vallate papilla up to end of the tongue) and anterior part of the tongue (from the tip of the tongue up to vallate papilla). Finally, one illustration represents the pharynx. At each location, the patient can indicate the severity of the perceived oral dryness using a 5-point Likert scale ranging from 1 = “no dryness” to 5 = “severe dryness” [9, 10].

### Sialometry and salivary pH

To limit circadian variations, the saliva measurements were performed between 8:15 and 10:15 A.M in the same room (temperature 20–24 ℃, humidity 50–70%) [28]. The participants were instructed not to eat, drink, chew gum, brush teeth, use mouthwash and smoke at least 1 h before their visit. The unstimulated (UWS) and chew-stimulated salivary flow rates (CH-SWS) were determined as described previously [29]. The pH of saliva was measured immediately after saliva collection using an electronic pH metre (PHM240, pH/ion metre, Meterlab, Copenhagen, Denmark). The samples were kept on ice until analysed.

### Determination of the palatal surface area

In order to measure the palatal surface area, an intra-oral scan of the upper jaw including the palate (the whole hard palate and part of the soft palate) was taken using a TRIOS 3 scanner (3Shape, version 21.3.5, Copenhagen, Denmark) using the manufacturer’s protocol. Scans were digitally saved as Polygon File Format (PLY) files.

Subsequently, each PLY object was analysed twice in Meshmixer (Autodesk, San Rafael, CA, USA) by one researcher (ZA). This analysis involved the manual separation of the palate by using the vibrating line including visible fovea palatine as a cut-off for the length of the palate. Besides, all palatal mucosa including the gingiva around the upper teeth were included in the palatal surface. After segmentation, the palatal surface areas (in mm^2^) were determined.

### Measuring the salivary film thickness

Determination of the salivary film was performed as described in previous studies [2, 11–14, 18–21]. Minimally 15 min after the collection of the whole saliva, the salivary film was collected at different intra-oral locations using Sialopaper filter paper strips (Oraflow, New York, USA). The filter strips were handled with gloved hands at all times. Five mucosal surfaces were selected based on previous studies [9, 10]: The anterior tongue was sampled in the middle of the tongue approximately 5 mm from the tongue tip, the anterior palate in the middle at the papilla incisive, the posterior palate in the middle at the vibrating line, the inside cheek 1 cm from the right chelion at the occlusal plane and the floor of the mouth at the right sublingual caruncula. The salivary film was collected twice at each location. Participants were instructed to swallow each time before a Sialopaper was applied to the surface for 5 s. The volume of fluid absorbed on the strip was measured electronically using a calibrated micro-moisture metre (Periotron 8000; Oraflow, Hewlett, NY, USA) and stored in Eppendorf tubes (Eppendorf, Cambridge, UK). Participants were instructed to swallow, and a second sample was collected at the same location. Samples were kept on ice until analysed. The salivary film thicknesses were calculated by dividing the collected saliva by the surface area of a Sialostrip (44.15 mm^2^).

### Measuring MUC5B levels

The MUC5B levels were determined essentially as described before [6, 30–34]. High-binding 96-well polystyrene microplates (Greiner Bio-One) were used for all ELISAs. The unstimulated saliva samples were vortexed for approximately 10 s and centrifuged (10 min, at 10.000 g). The supernatant was transferred to a new vial. Supernatants were diluted 1:200 in coating buffer (0.1 M NaHCO_3_, pH 9.6), and per sample 100 µL/well was coated in duplicate on the microplates.

MUC5B was eluted from the Sialopapers with MilliQ water (210 µL) with an efficiency of 84 ± 15% (data not shown) and then diluted in 210-µL coating buffer. Afterwards, eluted samples (100 µL/well) were coated in duplicate on the 96-well microplates, and all wells were serially diluted in coating buffer. Afterwards, all microplates were subsequently incubated at 37 ℃ for 2 h. Then the wells were rinsed with PBS–0.1% Tween 20 (PBST) for three times. The plates were then blocked for 1 h with 100 µL per well with 1% gelatin in PBST (PBSTG). After removing the blocking solution, 100 µL per well of 1:40 mAb F2, recognising the terminal part of the carbohydrate moiety, sulfo-Lewis-A SO3-3Gal_1-3GlcNAc in PBSTG [5, 30, 31, 33, 34]. The microplates were then incubated for 1 h at 37 ℃. After washing, the microplates were incubated for 1 h with rabbit-anti-mouse IgG-HRP conjugate (Rockland Immunochemicals Inc., Pottstown, PA, USA) 1:2000 in PBSTG. After washing with PBST and distilled water, 100 µL TMB solution (3,3’,5,5’-tetramethyl-benzidine; 125 µg/ml in sodium acetate buffer (100 mM, pH 5.0) with 0.05% v/v H_2_O_2_) was added to each well. After 10 min, the reaction was stopped by adding 50 µL 2 M H_2_SO_4_ per well. Absorbance was measured at 450 nm with a plate spectrophotometer reader (Multiskan FC, Thermo Scientific, Waltham, MA, USA). Arbitrary units (AU) MUC5B were calculated using a reference sample, as described before [6, 30, 35].

### Statistical analysis

The data were processed in an electronic clinical data-management platform (CastorEdc, Castor, Amsterdam, the Netherlands) and then converted into SPSS version 27.0 (IBM Corp SPSS Statistics, Armonk, NY, USA) for the statistical analysis. The Shapiro–Wilk test was used to assess the normality of the data. The data were presented as median and their interquartile range (IQR), as most of the parameters were not normally distributed. The mean and standard deviation were also reported to clarify relatively small differences.

The intraclass correlation coefficient (ICC) was used to determine the degree of agreement between two measurements for the palatal surface area. A two-way mixed, absolute agreement, average-measures ICC was calculated for these measurements [36, 37]. The ICC is indicative of poor (values less than 0.5), moderate (between 0.5 and 0.75), good (between 0.75 and 0.9) and excellent (greater than 0.90) reliability [38].

The mean of the two palatal surface area measurements, the two salivary film measurements and two MUC5B levels at each location were used for further analysis.

Female-male differences for various saliva characteristics, including the salivary flow rate, total XI-score and intra-oral RODI-scores, were explored with a Mann–Whitney U test.

A Friedman test was conducted for the salivary film thickness and the MUC5B levels at various intra-oral locations, followed by a Wilcoxon signed-rank test as a post-hoc procedure.

Various possible associations were explored in the current study. These relations were analysed with a bootstrapped Pearson correlation test (1000 × bootstrapping). The Pearson correlation coefficient and bias-corrected accelerated (Bca) 95% confidence interval were extracted. The following correlations were investigated: between the salivary film thickness with the MUC5B levels at the five corresponding intra-oral locations, between the salivary film thickness of the palate with the palatal surface area and between the MUC5B levels of the palate with the palatal surface area. Furthermore, the participants were dichotomized based on their sex and the dimensions of the palate. The median of the palatal surface area was used to create two equal groups: ‘small’ palatal surface area (< 2138.0 mm^2^) and ‘large’ palatal surface area (≥ 2138.0 mm^2^). The size of the correlation coefficient was interpreted as poor (*r* = 0.1–0.2), fair (*r* = 0.3–0.5), moderate (*r* = 0.6–0.7) or very strong (*r* = 0.8–0.9) correlation [39].

Furthermore, a multivariate analysis, multiple linear regression, was performed to investigate the possible association between the salivary film thickness and all independent variables. The salivary film thicknesses of both the anterior and the posterior palate were considered as dependent variables, while the palatal surface area, sex, the UWS and CH-SWS flow rate were considered as independent variables. All these independent variables were chosen because they could affect the thickness of the salivary film. To identify the degree of multicollinearity among the independent variables, the variance inflation factor (VIF) was calculated. The VIF for these variables was < 5, which indicates that there is no multicollinearity present among these variables [40, 41]. Additionally, the R square will be reported.

No multiple regression was conducted for the MUC5B levels of the anterior palate as the variance of the residuals was not constant and also multivariate normality was not met (residuals were not normally distributed).

All significance levels (*P*) were set at 0.05.

## Results

**Fig. 1:**
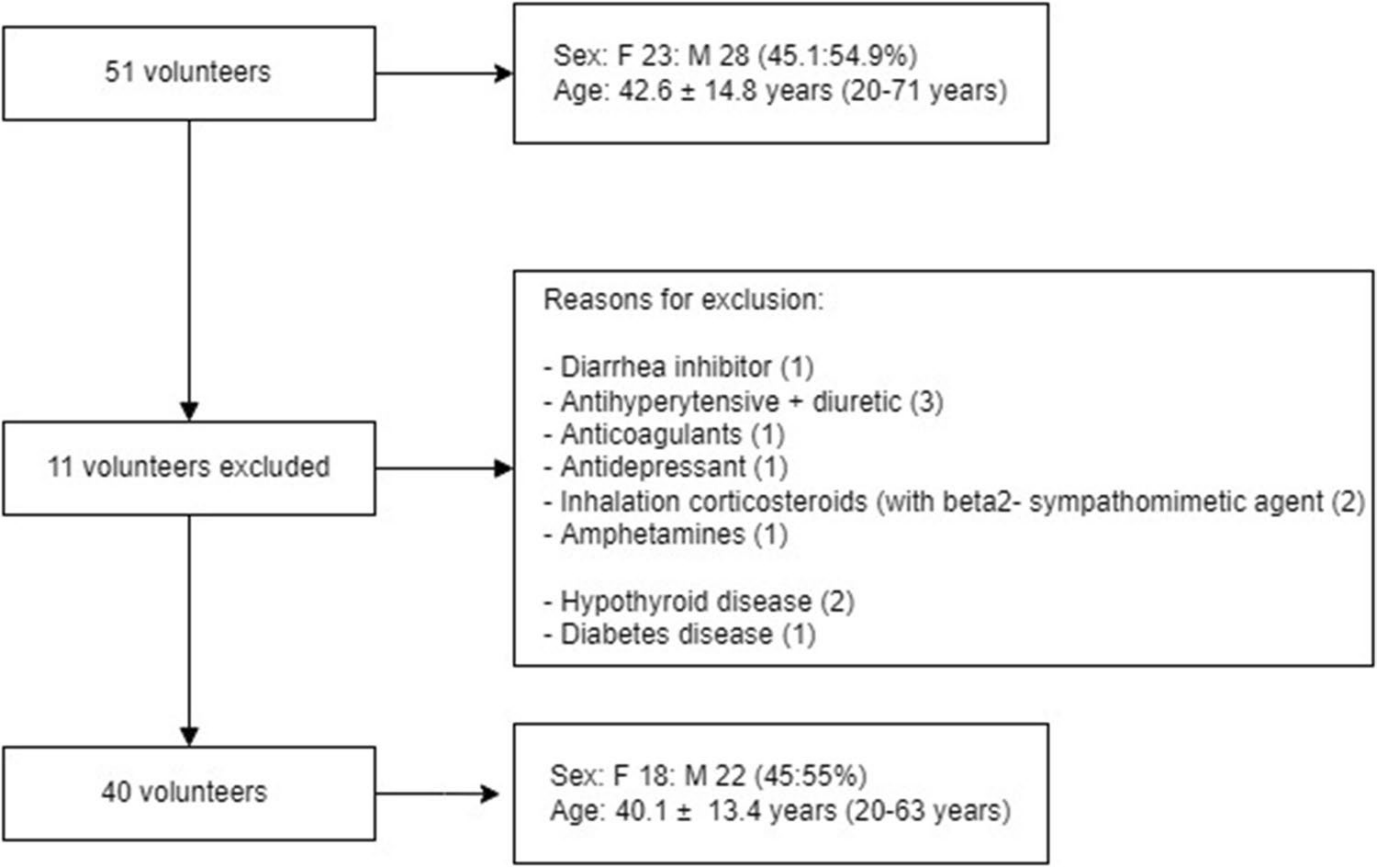
Flow chart showing the reason for exclusion of some volunteers and the characteristics of the included volunteers

Fifty-one volunteers signed up for this study (Fig. 1). The average age of female and male participants did not differ significantly (Mann–Whitney *U* test *p* > 0.05). Eleven volunteers had a systemic disease and/or were taking various medications that could initiate dry-mouth symptoms (Fig. 1). After the exclusion of these volunteers, the average age of the remaining 40 volunteers was 40.1 ± 13.4 years. The average age of the female and male participants did not differ significantly (Mann–Whitney *U* test *p* > 0.05). Ten of the remaining 18 female volunteers used contraceptive medication.

### Sialometry, salivary pH, dry-mouth experience and palatal surface area measurement

Table 1 reports the salivary secretion rates and pH, the overall dry-mouth experience as measured with XI and the palatal surface area measurement. The median UWS salivary flow rate for all participants was 0.25 ± 0.16–0.37 mL/min, while the median CH-SWS flow rate was approximately 5 times more than that of the UWS. The median salivary pH for the CH-SWS (pH = 7.14) was higher compared to UWS (pH = 6.60). Female and male participants did not show any significant difference with regard to the salivary flow rate and pH of both UWS and CH-SWS (Mann–Whitney *U* test *p* > 0.05).

**Table 1:** Characteristics of the total study population (40 volunteers) and stratified according to sex

Saliva		Total study population, mean ± SD	Total study population, median ± IQR	Females, median ± IQR (*N* = 18)	Males, median ± IQR (*N* = 22)
UWS	Flow rate	0.28 ± 0.15	0.25 ± 0.16–0.37	0.31 ± 0.16–0.43	0.23 ± 0.15–0.30
	pH	6.56 ± 0.29	6.60 ± 6.39–6.77	6.66 ± 6.45–6.82	6.59 ± 6.29–6.71
CH-SWS	Flow rate	1.26 ± 0.67	1.19 ± 0.74–1.58	1.30 ± 0.85–1.58	1.14 ± 0.72–1.61
	pH	7.12 ± 0.31	7.14 ± 6.91–7.33	7.15 ± 6.98–7.34	7.13 ± 6.82–7.32
XI-total		19.9 ± 5.2	19.5 ± 16.3–23.8	19.5 ± 17.8–24.3	19.5 ± 15.8–23.3
Surface area (mm^2^)					
Palatal	ICC = 0.96	2123.8 ± 221.9	2138.0 ± 1975.5–2247.6	2050.6 ± 1868.4–2177.9^*^	2176.8 ± 2018.9–2260.5

The total XI-scores, the unstimulated whole saliva (UWS), chewing-stimulated whole saliva (CH-SWS) flow rate (mL/min), salivary pH and palatal surface area (mm^2^) were reported. Data are expressed as medians with the corresponding interquartile ranges (IQR) and as means with standard deviations (SD). The ICC is reported only for the palatal surface area measurement

^*^Female vs. male difference Mann–Whitney *U* test *p*-value < 0.05

The median XI-score was 19.5 out of the maximum of 55 (Table 1). The XI-values for female and male participants also did not differ significantly (Mann–Whitney *U* test *p* > 0.05).

The intra-oral regions with the highest RODI-scores were the upper lip (M = 1.68 ± 0.86, Mdn = 1.00 ± 1.00–2.00), the posterior palate (M = 1.63 ± 0.74, Mdn = 1.50 ± 1.00–2.00), the lower lip (M = 1.60 ± 0.78, Mdn = 1.00 ± 1.00–2.00) and the pharynx (M = 1.60 ± 0.67, Mdn = 1.50 ± 1.00–2.00). In contrast, the floor of mouth had the lowest RODI-score (M = 1.10 ± 0.30, Mdn = 1.00 ± 1.00–1.00). The RODI-scores for all intra-oral locations were < 2, indicating that the volunteers did not experience any intra-oral dryness. Females and male participants did not differ significantly in RODI-scores for each of the intra-oral regions (Mann–Whitney *U* test *p* > 0.05).

The median palatal surface area was 2138.0 ± 1975.5–2247.6 mm^2^. The ICC for the surface area measurements was 0.96, which is in the excellent range. The palatal surface area showed significant differences for both sexes (Mann–Whitney *U* test *p* < 0.05), whereas male participants had a significantly larger palatal surface area compared to females (Table 1).

### Salivary film thickness and MUC5B levels at various intra-oral locations

The salivary film thickness showed considerable differences between the intra-oral locations. For example, the salivary film at the anterior tongue was six times thicker compared to that at the anterior palate (Table 2, Wilcoxon signed-rank tests: *p* < 0.05). Moreover, the salivary film thickness of the floor of the mouth differed significantly from all other intra-oral locations (Wilcoxon signed-rank tests *p* < 0.05). Besides, there was a significant difference in film thickness between the anterior and posterior palate, as the saliva film on the posterior palate was 2.6 times thicker compared to the film on the anterior palate (Table 2, Wilcoxon signed-rank tests: *p* < 0.05).

**Table 2:** The salivary film thickness at five intra-oral location, stratified according to sex

Intra-oral loacations	Salivary film thickness in µm (*N* = 40), median ± IQR	Female salivary film thickness in µm (*N* = 18), median ± IQR	Male salivary film thickness in µm (*N* = 22), median ± IQR
Anterior part of the tongue	68.9 ± 57.6–77.4	65.2 ± 57.4–73.4	74.0 ± 59.4–79.3
Anterior part of the palate	11.3 ± 5.2–19.1^a^	6.0 ± 4.1–13.8^**^	15.7 ± 9.2–22.0
Posterior part of the palate	29.7 ± 17.4–44.3^a,b^	28.0 ± 15.7–39.0	31.4 ± 21.1–53.3
Inside cheeks	44.0 ± 34.8–58.5^a,b,c^	40.0 ± 27.1–50.0^**^	55.4 ± 39.7–68.5
Floor of the mouth	62.5 ± 46.3–78.7^a,b,c,d^	52.5 ± 33.5–78.9	67.4 ± 54.4–78.6

*N* indicates the number of participants in each group. Data are presented as median with corresponding interquartile range (IQR)

^**^Female vs. male difference Mann–Whitney *U* test *p*-value < 0.01

^a^Wilcoxon signed-rank tests: *p* < 0.05 vs. anterior part of the tongue

^b^Wilcoxon signed-rank tests: *p* < 0.05 vs. anterior part of the palate

^c^Wilcoxon signed-rank tests: *p* < 0.05 vs. posterior part of the palate

^d^Wilcoxon signed-rank tests: *p* < 0.05 vs. inside cheeks

The salivary film thickness differed significantly between males and females only for the anterior palate and the inside cheeks, whereby the salivary film in these two regions was thicker for male participants.

Total unstimulated saliva contained the highest levels of MUC5B, *i.e.* 0.345 ± 0.177–0.716 AU/mL. The MUC5B levels in total saliva of female participants (0.369 ± 0.176–0.762 AU/mL, *N* = 18) did not differ significantly from male participants (0.331 ± 0.171–0.555 AU/mL, *N* = 22) (Mann–Whitney *U* test *p* > 0.05). Significant differences in MUC5B levels between the intra-oral locations were found (Table 3). The MUC5B level at the anterior tongue was 42 times higher than at the anterior palate, where the lowest level was measured. MUC5B level at the anterior palate showed significant differences between the two sexes, with female participants having lower MUC5B level than male participants (Mann–Whitney *U* test *p* < 0.05) (Table 3).

**Table 3:** The MUC5B levels at five intra-oral locations, stratified according to sex

Intra-oral locations	MUC5B in AU/mL (*N* = 34)^#^, median ± IQR	Female MUC5B in AU/mL (*N* = 16)^#^, median ± IQR	Male MUC5B in AU/mL (*N* = 18)^#^, median ± IQR
Anterior part of the tongue	0.127 ± 0.040–0.353	0.089 ± 0.037–0.173	0.273 ± 0.040–0.638
Anterior part of the palate	0.003 ± 0.000–0.011^a^	0.000 ± 0.000–0.003^*^	0.006 ± 0.000–0.017
Posterior part of the palate	0.020 ± 0.009–0.121^a,b^	0.018 ± 0.005–0.039	0.027 ± 0.012–0.208
Inside cheeks	0.008 ± 0.000–0.034^a,b^	0.008 ± 0.000–0.032	0.010 ± 0.000–0.075
Floor of the mouth	0.007 ± 0.000–0.029^a,b,c^	0.002 ± 0.000–0.030	0.012 ± 0.000–0.029

*N* indicates the number of participants in each group. Data are presented as median with corresponding interquartile range (IQR)

^*^Female vs. male difference Mann–Whitney *U* test *p*-value < 0.05

^a^Wilcoxon signed-rank tests: *p* < 0.05 vs. anterior part of the tongue

^b^Wilcoxon signed-rank tests: *p* < 0.05 vs. anterior part of the palate

^c^Wilcoxon signed-rank tests: *p* < 0.05 vs. posterior part of the palate

^#^The total number differs as MUC5B samples were not available for all participants

### Association between salivary film thickness and the MUC5B levels

The salivary film thickness of all intra-oral locations showed significant correlations with the MUC5B levels of the associated regions (Table 4). The correlation coefficients varied between 0.48 and 0.66, which can be considered as fair to moderate. A positive correlation indicates that when MUC5B levels increase, the salivary film thickness at the associated region is also increased. Only the floor of the mouth did not have any significant correlation between the salivary film thickness and MUC5B levels for the total study population. However, when this group was stratified on sex, it was found that females had a significant correlation for the floor of the mouth. For all other intra-oral regions, it was found that the correlation coefficient of both sex groups lied in the same range as the total study population. Only females did not have any significant correlation between the salivary film thickness and the MUC5B levels at the anterior tongue (Table 4).

**Table 4:** The correlation between the salivary film thickness at five intra-oral regions with the MUC5B level at the associated regions

Correlation between film thickness and MUC5B levels at associated regions	Correlation coefficient (total study population)	Correlation coefficient (females)	Correlation coefficient (males)
Anterior part of the tongue	0.57 (0.42–0.74)^**^	NS	0.63 (0.34–0.84)^**^
Anterior part of the palate	0.66 (0.46–0.86)^**^	0.57 (− 0.06–0.89)^*^	0.63 (0.33–0.87)^**^
Posterior part of the palate	0.56 (0.33–0.78)^**^	0.61 (− 0.11–0.90)^*^	0.59 (0.24–0.90)^**^
Inside cheeks	0.48 (0.21–0.75)^**^	0.67 (0.08–0.85)^**^	0.54 (0.14–0.85)^*^
Floor of the mouth	NS	0.52 (0.18–0.82)^*^	NS

Data are expressed as the Pearson correlation coefficient and bias-corrected accelerated (Bca) 95% confidence interval

*NS*, not significant

^*^Pearson correlation test *p*-value < 0.05

^**^Pearson correlation test *p*-value < 0.01

### Association between the salivary film thickness and the MUC5B levels at the palate with the palatal surface area

The salivary film thickness and MUC5B levels at the anterior and posterior palate did not have any significant correlation with the palatal surface area (Pearson correlation *p* > 0.05). Because male participants had a significantly larger palatal surface area, this analysis was repeated after stratifying the participants based on their sex. The palatal surface areas of both female and male did not have any significant correlation with the salivary film thickness and/or MUC5B levels of the anterior and posterior palate (Pearson correlation *p* > 0.05). Besides, the two palatal dimensions (small vs. large surface area) did not have any significant correlation with the salivary film thickness and/or MUC5B levels of the palate as well (Pearson correlation *p* > 0.05).

A multivariate regression analysis was performed, taking the palatal surface area, sex, the UWS and CH-SWS flow rate into consideration. For both the anterior and posterior salivary film thickness, no association was found with any of the independent variables (regression *p* > 0.05). The *R* squared for the anterior palate was 0.19 and for the posterior palate 0.09. So, the palatal surface area did not affect the salivary film thickness on both the anterior and posterior palate. The same applied to all other independent variables.

## Discussion

The results of this study, in which we explored the salivary film thickness and MUC5B levels at various locations in the oral cavity in healthy volunteers, demonstrated that both are unequally distributed over the various intra-oral surfaces. The anterior tongue had the thickest salivary film and contained the highest levels of MUC5B, while the anterior palate had the thinnest salivary film and lowest MUC5B levels. Furthermore, the palatal surface area did not correlate with the palatal salivary film thickness or the palatal MUC5B levels, indicating that in healthy individuals, a larger surface area was not associated with a relatively thinner salivary film and/or lower MUC5B levels. Therefore, our hypothesis should be rejected.

The mean UWS flow rate of the included participants was 0.28 mL/min, which was comparable with the average values of 0.3–0.4 mL/min previously reported [42].

The median XI-score was 19.5, indicating that included participants did not experience serious dry-mouth complaints. The current XI-scores were comparable with the XI-scores found in other studies with healthy volunteers (age from 18 to 92 years), varying between 16.0 and 20.82 [43–49]. Also, the RODI-scores for all intra-oral locations were < 2, indicating they did not experience any intra-oral dryness. Dry-mouth patients in previous studies showed RODI-scores ≥ 3 for most intra-oral locations [9, 10]. So, although the salivary flow rate seems to deviate slightly from earlier reports, it can be stated the included volunteers had healthy salivary flow rates and experienced no dry-mouth complaints.

The average palatal surface area found was 2123.8 mm^2^, which was comparable with other studies, who included adults with an average of 1990–2010 mm^2^ [15, 16, 50]. In these studies, the palatal surface areas were determined using foil impressions taken from stone models, while another study used CBCT imaging and digital analysis [15, 16, 50]. Apparently, all methods used so far reveal comparable and representative results as their surface areas are in the same range. In addition, the technique presented in the current study, using an intra-oral scanner, adds up to this line of methods as it had very good reproducibility, as indicated by the excellent range of the ICC. However, future studies, which investigate and compare the validity and the reliability of various methods including the intra-oral scanner for measuring the intra-oral surface area, seem warranted.

The pattern of salivary film distribution over intra-oral locations found in the current study was comparable with the distribution of the salivary film in healthy volunteers reported previously [2, 11–14, 18–21]. Also, comparable patterns were seen in the current study, as the tongue and/or the floor of the mouth had the thickest salivary film, while the anterior palate had the thinnest salivary film. The reason why the tongue has the highest level of wetness is probably because of its anatomical location near the caruncle of the Wharton’s ducts [2, 13, 18]. Here, saliva from the many minor glands in this region and the nasopalatine glands as well as the secretions of the submandibular and sublingual glands is collected [2]. Besides, the von Ebner’s glands, with their ducts opening into the sulci of the circumvallate and foliate papillae, produce serous saliva that contributes to the moistening of the tongue [51, 52]. In contrast, several factors make the anterior palate more susceptible to having a thin salivary film compared to other intra-oral locations; lack of hard palatal salivary glands and evaporation, especially during speaking and breathing [18, 53, 54]. Besides, gravity forces part of the excreted saliva to pool on the floor of the mouth between swallowing episodes. As a consequence, the palate can be moistened with less sufficiently [2].

Two previous studies investigated MUC5B levels at various intra-oral locations in healthy controls [11, 14]. However, different techniques were used in these studies compared to our study: Firstly, SDS-PAGE was performed on the eluted Sialopapers with subsequent PAS staining. Then, software analysis was used, scanning lanes of PAS-stained mucin glycoprotein bands, and analysed for colour intensity, gauging the amount of mucin [11, 14]. In contrast, we applied ELISA using an antibody, i.e. F2, to specifically measure MUC5B levels. However, it seemed difficult to compare our findings to those of Chaudhury et al*.* [11] because they expressed the MUC5B levels in MUC5B glycan/protein proportion. In contrast, we calculated arbitrary units/volume of fluid on Sialopaper [11]. In the study by Pramanik et al*.*, contradictory results compared to our study were found; they found the highest MUC5B levels at the anterior hard palate and the lowest levels at the lower labial mucosa and the anterior tongue [14]. In contrast, in our study, the anterior tongue had the highest levels, and the anterior palate had the lowest levels of MUC5B. This is difficult to explain as to a large extend MUC5B is secreted by the submandibular and sublingual salivary glands with their sublingual caruncle lying on the floor of the mouth [55, 56], in which the tongue is embedded. As mentioned before, the anterior hard palate lacks the presence of salivary glands [22], and MUC5B found on the anterior palate is translocated there mainly by tongue movements.

Surprisingly, the floor of the mouth contained approximately 18 times less MUC5B levels compared to the anterior tongue, despite the fact that the caruncle of both submandibular and sublingual glands are located on the floor of the mouth. Gravity forces help the floor of the mouth to create a reservoir for all the saliva that does not adhere to the various surfaces. So, the saliva on the floor of the mouth is a mix of various salivary glands. Especially after swallowing episodes, not all the saliva is swallowed; the salivary clearance is approximately 28% [57, 58], indicating that the majority of saliva remains in the mouth. Additionally, the structure of the tongue helps to adhere to all the mucins, as the dorsal (superior) surface has a rough structure of stratified squamous epithelium with numerous circumvallate, filiform and fungiform papillae. Potentially, this rough or plicated surface offers the MUC5B glycoprotein a surface to which it can reside more effectively during oro-facial movements, such as swallowing, than to the smooth structure of the floor of the mouth.

An interesting finding in the current study was the significant correlation between the salivary film thickness and the MUC5B levels. MUC5B forms hydrophilic polymer brushes causing water retention [59]. For this reason, MUC5B is considered as the key lubricant in saliva. So, it could be expected that increasing MUC5B levels will influence the increment of the salivary film thickness.

Another interesting finding was the lack of correlation between the palatal surface area with the palatal salivary film thickness and/or the palatal MUC5B levels. We hypothesised that individuals with a larger palatal surface area would have a thinner salivary film at the palate and also less availability of MUC5B glycoproteins. However, we found that all individuals showed comparable salivary film thickness and MUC5B levels. This last result could be explained by the palatal saliva that contained relatively high levels of MUC5B [60]. Palatal saliva is excreted by the orifices of the palatal glands, which are all located at the right and left maxillary second and third molars [22]. The palatal saliva including MUC5B is propelled towards the anterior part of the palate during swallowing; this can possibly explain why the salivary film thickness and the MUC5B level are not particularly low in individuals with larger palatal dimensions. Additionally, the palatal salivary film is not only formed by the palatal salivary glands, but it is also dependent on the salivary film of the tongue. The tongue also plays an important role in moistening and lubricating the palate. As the salivary film thickness at the tongue is already 2.3–6 times thicker compared to the palate, this will promote the transfer of additional saliva from the tongue to the palate. Finally, the retainment of saliva by the anterior palate plays also a possible role. The structural orientation of the anterior palate, especially of the rugae with their irregular, asymmetric ridges [61], causes the retainment of mucins and moist despite the negative effect of gravity.

A possible limitation of the current study is the use of Sialopapers for the collection of MUC5B. Although the elution efficiency of MUC5B out of the Sialopapers is good (84 ± 15%), it has to be noted that the absorption of all MUC5B glycoproteins from the mucosal surfaces to the Sialopaper seems virtually impossible. Namely, the oral mucosal surfaces are more or less covered with a double layer: a lower surface-bound layer, which is the mucosal pellicle, and an upper salivary film, loosely attached to the mucosal pellicle [4]. It is plausible to assume that the efficiency of absorption of MUC5B from the loosely attached salivary layer to the Sialopaper is probably more effective compared to MUC5B from the mucosal pellicle. In this light, it also has to be noted that oral epithelial cells express membrane-bound mucin (MUC1), which can interact with MUC5B to develop the mucosal pellicle [4]. Consequently, this interaction hinders the adsorption of MUC5B of the mucosal pellicle to the Sialopaper. Transmission Electron Microscopy and immunogold labelling could be applied to study these interactions and shed light on the absorption efficiency [4]. These techniques already have successfully been applied for buccal epithelial cells, but not for other intra-oral surfaces [62].

A recent study revealed that the intra-oral scanner was a suitable instrument to investigate the palatal soft tissue in terms of shape, colour and curvature [63]. In line with our experience, the shape of the palatal surface, especially the palatal rugae, was documented very precisely with the intra-oral scanner. Yet, it has to be noted that the intra-oral scanner lacks the resolution to analyse the full microstructure of the palatal surface, which could lead to a slight underestimation of the total palatal surface area determined in the current study.

## Main conclusions

The salivary film and MUC5B levels were not equally distributed over the mouth. The anterior tongue had the thickest salivary film and also the highest levels of MUC5B, while the anterior palate had the thinnest salivary film and lowest MUC5B levels. There was no association between the palatal surface area and the salivary film thickness at the palate, also when sex and salivary flow rate were taken into consideration. These results indicate that a larger surface area is not associated with a relative thinner salivary film.
